# A150 DEVELOPMENT OF MIMETIC MATRICES AS INSTRUCTIVE ORGANOIDS MICROENVIRONMENT FOR THE STUDY OF COLONIC DISEASES

**DOI:** 10.1093/jcag/gwad061.150

**Published:** 2024-02-14

**Authors:** V Bat, C Jones, F Boudreau, N Faucheux, N Perreault

**Affiliations:** Universite de Sherbrooke Faculte de Genie, Sherbrooke, QC, Canada; Universite de Sherbrooke Faculte de Medecine et des Sciences de la Sante, Sherbrooke, QC, Canada; Universite de Sherbrooke Faculte de Medecine et des Sciences de la Sante, Sherbrooke, QC, Canada; Universite de Sherbrooke Faculte de Genie, Sherbrooke, QC, Canada; Universite de Sherbrooke Faculte de Medecine et des Sciences de la Sante, Sherbrooke, QC, Canada

## Abstract

**Background:**

The functional complexity of the colonic epithelium is dependent on its interaction with the microenvironment involving gradients of soluble factors, extracellular matrix (ECM) proteins and stiffness. The influence of dynamic changes in the ECM has been observed in tumours, with variations in protein expression and rigidity. Their biomolecular impacts on epithelial cell behaviour are less studied due to the structural complexity of the matrix. Organoids offers a novel approach for the study of ECM biodynamics, as they require a matrix (Matrigel) to develop. Previous work showed that it is possible to grow organoids in hydrogels other than Matrigel. To evaluate the effect of matrix composition alteration on cancer initiation, we developed biofunctionalized composite hydrogels that mimic the ECM changes (chemical and physical) observed in the *BmpR1a*-deficient telocytes^ Foxl1 +^ (*Bmpr1a*^△Foxl1+^) mouse model of colorectal cancer (CRC) initiation.

**Aims:**

The development of mimetic matrices, in association with organoids, would enable us to uncover the impact of matrix deregulation on colonic cells behaviour in pathogenesis process.

**Methods:**

These hydrogels were made of alginate and 8-arm polyethylene glycol- vinyl sulfone (PEG) macromers bearing cysteine residues (CYS) and peptides. Four peptides derived from fibronectin, laminin 111, collagen I and collagen IV were used to mimic the ECM changes in the *Bmpr1a*^△Foxl1+^ colon. The grafting of CYS on alginate or peptides on PEG macromers was first characterized by thiol quantification assays. The non-grafted peptides were also analysed by uHPLC. Stiffness of the hydrogels was then determined by dynamic mechanical analysis. Finally, normal mouse organoids were seeded on hydrogels and evaluated for survival by live/dead labeling and proliferation by Alamar blue assays. Matrigel was used as a control.

**Results:**

Chemical characterization revealed that CYS were grafted onto the alginate chain at around 190 μmol/g alginate. The PEG macromers were functionalized with four peptides at concentration varying from 0.25 to 1.5 mM. Rigidity of the hydrogels depends on the concentration of alginate used where lower concentration leads to soft matrix (0.5 kPa, homeostasis) and high concentration produced a stiff matrix (4 kPa, diseased). Organoids seeded in these mimetic composite hydrogels survived and proliferated in comparison to alginate alone.

**Conclusions:**

Our results indicate that we can develop mimetic composite hydrogels where colon organoids can survive and thrive. This is a breakthrough model for defining the roles of ECM mechanical and biochemical stimuli in epithelial behaviour during the initiation and development of CRC.

**Funding Agencies:**

CIHR

